# Case Report: Persistent left superior vena cava: an incidental finding during peripherally inserted central catheter placement

**DOI:** 10.3389/fsurg.2023.1254937

**Published:** 2024-01-03

**Authors:** Shi-Xiang Pan, Ye-Ling Zhang, Fang Fang

**Affiliations:** Department of Cancer Therapy, The Affiliated Hospital of Qingdao University, Qingdao, China

**Keywords:** chemotherapy, peripherally inserted central catheter, ECG, persistent left superior vena cava, enhanced CT

## Abstract

**Background:**

A peripherally inserted central catheter (PICC) is a specialized type of long-term intravenous catheter commonly employed for administering chemotherapy. The operation and management of PICC should exclusively be carried out by trained healthcare professionals equipped with the requisite knowledge of anatomy, procedural technique and patient care. Persistent left superior vena cava (PLSVC) is a vascular malformation which is typically asymptomatic in clinical presentation, almost always identified during invasive surgery or imaging examinations.

**Case presentation:**

Herein, we detailed a case involving a breast cancer patient whose PLSVC was identified during the placement of PICC because of the negative P-wave in electrocardiogram (ECG). Subsequent examination, including chest x-ray imaging, postoperative enhanced CT of the chest, ECG, and consultation with an experienced imaging physician confirmed that the patient's variant type was PLSVC type I. 2160. Removal of the catheter was deemed unnecessary, as the catheter tip was appropriately positioned and no other concomitant cardiovascular malformations were detected.

**Conclusion:**

The PLSVC is a vascular anomaly and is relatively uncommon within the general population. The operator should possess a thorough familiarity with the potential anatomical variations of left superior vena cava, and specialized case profile should be established for patients diagnosed with PLSVC.

## Introduction

Cancer treatment approaches continue to evolve and optimize, including operation, chemotherapy, radiation therapy, etc. (1). Among these methods, chemotherapy holds a significant role, often integrated with other treatments to provide a comprehensive strategy. Chemotherapy agents can be administered through diverse routes, including oral administration, intravenous infusion, injections (subcutaneous or intramuscular), or even topical applications (creams or gels). A peripherally inserted central catheter (PICC) stands as a specialized type of long-term intravenous catheter, extensively employed for administering chemotherapy and other medical therapies (2). A PICC provides medium- to long- term central venous access, granting direct entry to larger blood vessels for dispensing chemotherapy agents, fluids, nutrition, and medications.

The operation and management of a PICC should exclusively be carried out by trained healthcare professionals who possess essential expertise in anatomy, procedural technique, and patient care (3). It is utmost importance to understand anatomical abnormality for the operator which can occur arise from genetic factor, developmental issue, congenital condition, or acquired disorder (4). In this context, we presented a case involving the finding of persistent left superior vena cava (PLSVC) during PICC placement.

## Case presentation

**Figure 1:**
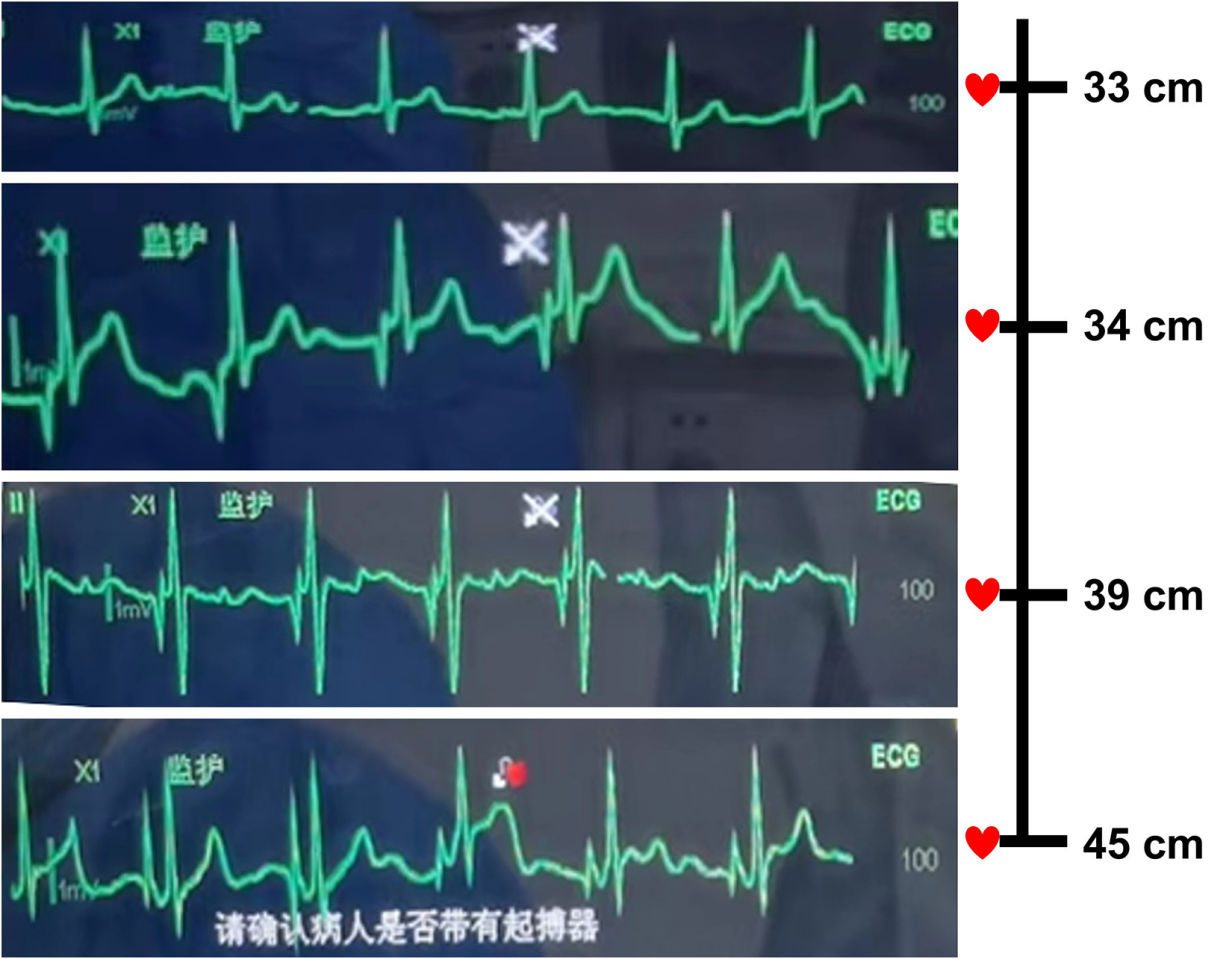
Electrocardiogram manifestation during the process of advancing and retreating the catheter. P-wave changes were dynamically observed on lead II of IC-ECG. IC-ECG showed that, at 33 cm, sinus rhythm with positive P-waves; at about 34 cm, negative P-waves instead of positive P-waves appeared. As the catheter continued to advance, the amplitude of the negative P-wave gradually increased and reached the maximum, and then the P-wave began to become biphasic and the amplitude decreased. At 45 cm, the negative P-wave disappeared completely. This phenomenon occurred repeatedly with repeated insertions of the catheter.

**Figure 2:**
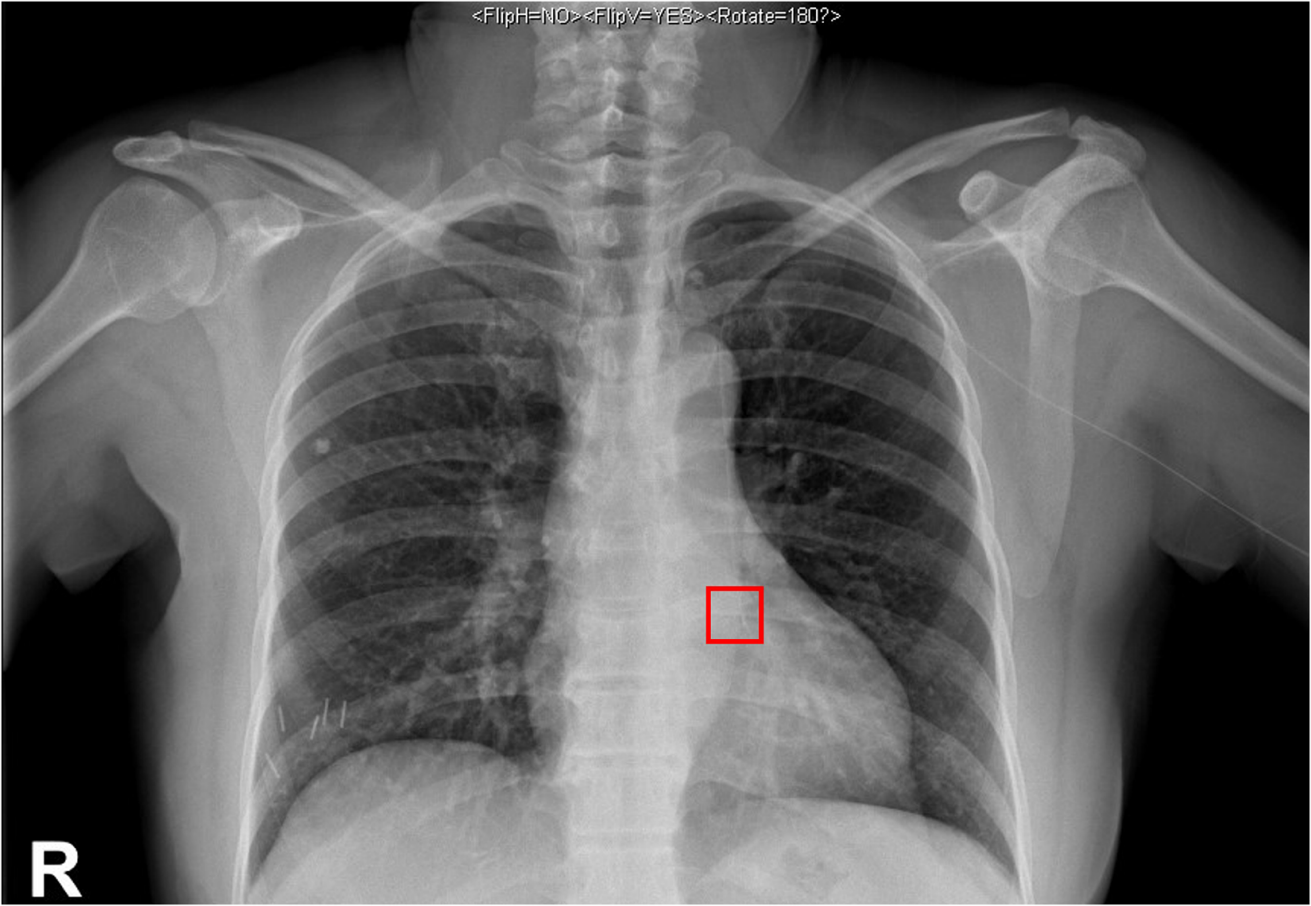
Chest x-ray findings. A chest x-ray was taken to confirm the tip location and showed that the catheter was positioned at the left paramediastinal border near the T8 vertebrae.

**Figure 3:**
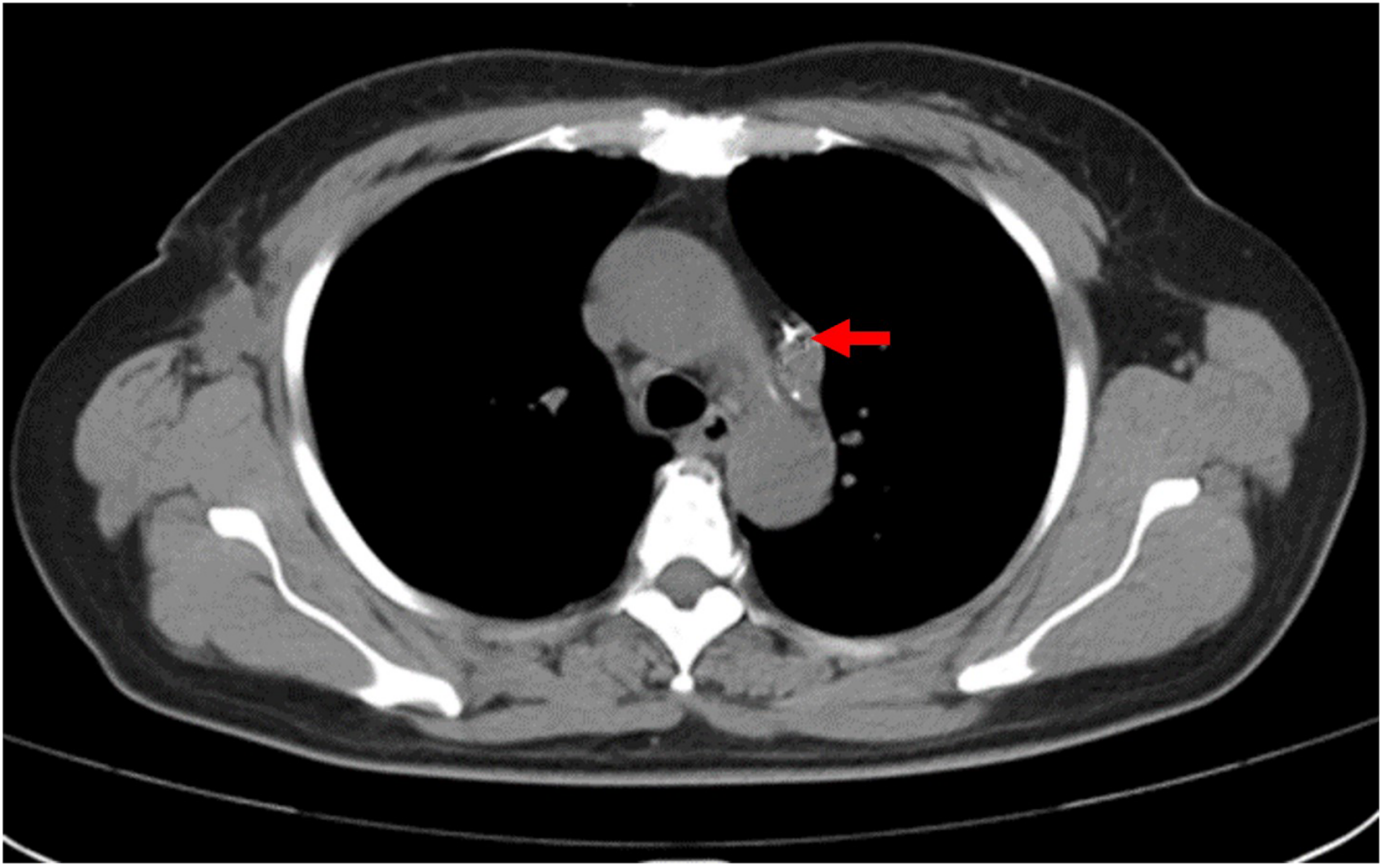
The postoperative enhanced CT of the chest. The postoperative enhanced CT of the chest showed that the diameter of the left superior vena cava is 11 mm, and compare the inner diameters of the PLSVC with the superior vena cava to ensure that the PLSVC could function normally.

A 64-year-old female patient was admitted to the hospital due to a 2 cm diameter tumor identified in her right breast. The pathology report from fine-needle aspiration indicated invasive ductal carcinoma in the right breast, grade 2. Subsequently, the patient underwent a successful right breast-conserving surgery along with a standard axillary dissection involving with sentinel node biopsy. The operation was effective and the patient's recovery had been favorable. A PICC placement was scheduled for subsequent postoperative chemotherapy. Prior to the operation, a comprehensive assessment of the patient's blood vessels and blood examination were performed. Throughout the operation, electrocardiogram (ECG) was employed to monitor vital signs and the positioning of the catheter tip. The patient was positioned supine, with the left arm abducted at a 90° angle from the body. Using B-ultrasound guidance, the puncture site on the left basilic vein was identified and marked on the body surface. The distance from the puncture site to the third intercostal space, down to the right sternoclavicular joint, was measured to provide an initial estimate of the catheter length. Following a thorough disinfection of the entire left arm using chlorhexidine and maintaining aseptic conditions, the basilic vein was punctured and the catheter inserted under B-ultrasound guidance. While advancing and retracting the catheter, dynamic monitoring of P-wave changes was conducted on lead II of the ECG. The ECG revealed a distinct deviation from the typical right superior vena cava (SVC) catheterization pattern. Specifically, at around the 34 cm, negative P-waves emerged instead of the expected positive P-waves. As the catheter continued its advance, the amplitude of these negative P-waves gradually intensified, eventually peaking before transitioning into a biphasic pattern with diminishing amplitudes. Around the 45 cm, the negative P-wave phenomenon ceased entirely. This abnormal phenomenon occurred repeatedly with repeated insertions of the catheter (Figure 1). A chest x-ray was taken to reconfirm the tip location and revealed that the catheter was positioned along the left paramediastinal border in proximity to the T8 vertebrae, rather than crossing the midline to access the right SVC (Figure 2). The subsequent postoperative enhanced CT demonstrated that the PLSVC could function normally within the SVC (Figure 3). The correlation of findings from chest x-ray imaging, postoperative enhanced CT, ECG, and consultation with a proficient imaging physician collectively confirmed the patient's specific anatomical variant as PLSVC type. 2160;. The vascular path was as follows: left upper brachial vein—left subclavian vein—PLSVC—CS—RA.

After the acquisition of informed consent from both the patient and family, we re-adjusted the catheter tip position with the guidance of ultrasound. As the catheter neared the RA, it was gently retracted by approximately 3 cm, ensuring that the PICC's tip was appropriately lodged within the lower third of the left SVC. Moreover, a special case file was established emphasizing the catheter maintenance. The patient and her family received unique health education about the PICC. The patient was requested to visit the PICC specialist Outpatient Department weekly during chemotherapy period. The tip position was verified by x-ray imaging before each chemotherapy. Beside from routine maintenance, the left arm circumference, the length of the external portion of the PICC was measured, and any symptoms of the patient were documented. The patient experienced an uneventful recovery following six cycles of chemotherapy. The dwell time of this PICC was 192 days.

## Discussion

Cancer treatment approaches have constantly evolved and been optimized over recent decades, encompassing different conventional and emerging therapies such as operation, chemotherapy, radiation therapy, and more. Among these, chemotherapy, involving the use of drugs to eliminate or impede the growth of cancer cells, stand as an important treatment option for cancer and is frequently integrated with other therapeutic methods to make a comprehensive strategy. Chemotherapy agents can be administered orally (pills or capsules), intravenously, or through other methods such as injections or topical applications. A PICC has been recognized as a favourable protocol for venous access in cancer chemotherapy.

A PICC involves the insertion of a catheter at a peripheral site (such as the arm), which is then advanced until its tip reaches the SVC just above its junction with the RA (2). This procedure can be performed either at the bedside or in a specialized intravenous room by trained individuals, including anesthesiologists, interventional radiologists, pediatricians, or specialized nurses (5). Given its path into the vena cava, a PICC dwelling time can be 390–575 days for infusion and blood sampling (2). By minimizing the need for repeated peripheral venous insertions, a PICC can significantly alleviate patient discomfort, pain, and anxiety. PICC-associated complications, such as thrombosis, stenosis, and infection have also been reported at relatively low levels (6). Therefore, PICC is the preferable choice for achieving intermediate to long-term venous access for medication, fluid therapy, blood sampling, and parenteral nutrition.

It is essential to be well-acquainted with the common anatomical variations in the venous system because they may lead to unusual discoveries either during PICC placement or on subsequent chest imaging. PLSVC is a vascular anomaly, in patients with which less research was reported about PICC implanted. The PLSVC is because of the persistence of the left anterior cardinal vein and the left common cardinal vein during embryonic development, which result in a persistent connection between the left brachiocephalic vein and the RA (7). The prevalence of PLSVC in the general population is approximately 0.3% to 0.5% and up to 10% among individuals with congenital heart disease (Table 1) (8). Typically asymptomatic, this malformation is often discovered incidentally, such as during the positioning of a central venous catheter placed from the left side of the body. In this study, we present a case involving the identification of PLSVC during the placement of PICC. The shared experience of this patient aimed to provide insights into variations in performing PICC for patient with this condition and highlight awareness about the importance of thoroughly examining venous anatomy before the procedure for patient safety and procedure success.

**Table 1:** The cases about PLSVC published in recent years.

Age	Gender	Primary Disease	Management	Author	Title	Journal	Year
61 Years	Female	Septic shock with acute renal failure	Continuous renal replacement therapy	Elke Schwier, et al.	Left-sided superior vena cava	Canadian Journal of Anaesthesia	2023
68 Years	Male	Coronary artery bypass graft surgery	Coronary artery bypass graft surgery	Steven Imburgio, et al.	Persistent Left Superior Vena Cava: An Illustrative Example	Journal of Cardiothoracic and Vascular Anesthesia	2023
79 Years	Male	Septic shock.	Hemodialysis catheter placement	Hemodialysis catheter, et al.	Isolated Persistent Left Superior Vena Cava	The New England Journal of Medicine	2022
35 Years	Male	Chronic kidney disease	Hemodialysis catheter insertion	Tuncay Sahutoglu, et al.	Persistent left superior vena cava: Two case reports and a review from nephrologists' perspective	Hemodialysis International	2016
80 Years	Male	Renal failure	Renal replacement therapy	Tuncay Sahutoglu, et al.	Persistent left superior vena cava: Two case reports and a review from nephrologists' perspective		
56 Years	Male	Superior left pulmonary lobe tumor	Routine chest x-ray	Cornel Savu, et al.	Persistent Left Superior Vena Cava—Accidental Finding	In Vivo	2020
22 Years	Female	Right lung pneumonectomy for chronic lung infection	Pre-surgical work-up, Transthoracic echocardiogram	Khadija Laasri, et al.	Persistent left superior vena cava: Case report	Radiology Case Reports	2022
40 Years	Male	Mixed Hodgkin's lymphoma	Totally implantable vascular access device	Etienne El-Helou, et al.	Persistent Left Superior Vena Cava Associated with Right Aberrant Subclavian Artery Detected during Totally Implantable Vascular Access Device Insertion	The Surgery Journal	2022
62 Years	Female	Hepatic failure	Preoperative transesophageal echocardiography	Taehee Kim, et al.	Clinical Implications of a Persistent Left Superior Vena Cava in a Patient With Right Superior Vena Cava Thrombosis Undergoing Emergency Deceased Donor Liver Transplantation: A Case Report	Transplantation Proceedings	2022
41 Years	Male	End-stage renal disease	Central venous catheterization	Osama A Samara, et al.	Isolated Persistent Left Superior Vena Cava Associated With Autosomal Dominant Polycystic Kidney Disease (ADPKD): Challenges and Clinical Significance	Cureus	2022

Based on distinct hemodynamic characteristics, PLSVC includes the following types (9, 10). In type I, the PLSVC drains into the RA through the CS, constituting approximately 90% of all PLSVC cases. When the catheter tip is correctly positioned, it can continue to be used because these patients have no other cardiovascular abnormalities (11). In type Ⅱ, similar to type I, the PLSVC also drains into the RA through the CS, however there is an additional patent connection to the left atrium, resulting in a right-to-left shunt. In type Ⅲ, the PLSVC directly drains into the left atrium and leads into right-to-left shunt. In type Ⅳ, CS atresia is observed, where the coronary sinus is blocked or non-existent. PLSVCs of Types II, III, and Ⅳ often exhibit varying degrees of cyanosis due to venous blood entering the left atrium and mixing with arterial blood (12). For patients with these three types of PLSVCs, the catheter should be removed (13). And these patients, who might have symptoms, such as cyanosis, syncope, reduced exercise tolerance, and progressive fatigue because of significant right to left cardiac shunting, should be referred to cardiothoracic surgery to receive treatments in two ways based on anatomy: PLSVC can be ligated if there is an adequate sized bridging vein, and PLSVC can be re-anastomosed to the CS if the bridging vein is not adequate in size or there is no right superior vena cava (9, 14).

For patients who had a recurring negative P-wave in lead Ⅱ of ECG during catheterization or for those patients whose catheter is positioned along the left side of the mediastinum on chest x-ray examination after catheterization, the possibility of PLSVC should be considered. This is attributed to the catheter's enter into the PLSVC and subsequently stimulates electrical impulses in advance, which is opposite to that of lead Ⅱ in body surface ECG; resulting in a negative P-wave appearing in lead II of the body surface ECG (15). And this negative P-wave, with a broad shape, is different from that occurs when the catheter enters the RA in patients with normal right SVC. As the catheter advances further, the amplitude of the negative P-wave diminishes, transitioning the P-wave into a biphasic configuration. This signifies that the catheter has now traversed the CS to enter the RA.

Catheter tip location is one of the primary focal points of the PICC procedure. During the placement of the catheter, when the maximum negative P-wave appears in the ECG, the catheter tip should be gently drawn back and fixed until the P/R ratio is approximately 50% of the maximum negative P-wave. This step aims to position the catheter tip accurately within the middle to lower segment of the PLSVC (16, 17). The PLSVC patient in this study was catheterized following the above-mentioned protocol, and subsequent confirmation of the catheter tip's accuracy was achieved through a chest CT scan conducted post-catheterization. The vital signs and clinical manifestations of the patients should be carefully observed during PICC operation, and timely identification and appropriate management of any complications related to the PICC are paramount. Finally, a special case file should be established to ensure safe PICC use in patients with PLSVC.

## Conclusion

In conclusion, the PLSVC is a vascular anomaly and is relatively uncommon within the general population. The operator should possess a thorough familiarity with the potential anatomical variations of left superior vena cava, and specialized case profile should be established for patients diagnosed with PLSVC.

## Data Availability

The original contributions presented in the study are included in the article/Supplementary Material, further inquiries can be directed to the corresponding author.
